# Integrating Multimorbidity Assessment into Rheumatology Care: Prognostic Role of the Charlson Comorbidity Index in Systemic Lupus Erythematosus

**DOI:** 10.3390/healthcare13182285

**Published:** 2025-09-12

**Authors:** Ryuichi Ohta, Yoshinori Ryu, Chiaki Sano, Kunihiro Ichinose

**Affiliations:** 1 Department of Community Care, Unnan City Hospital, Unnan 699-1221, Japan; yoshiyoshiryuryu.hpydys@gmail.com; 2 Community Medicine Management, Shimane University Faculty of Medicine, Izumo 693-8501, Japan; sanochi@med.shimane-u.ac.jp; 3 Department of Rheumatology, Shimane University Faculty of Medicine, Izumo 693-8501, Japan; kichinose@med.shimane-u.ac.jp

**Keywords:** systemic lupus erythematosus, Charlson comorbidity index, multimorbidity, risk stratification, prognosis, rheumatology care

## Abstract

**Background/Objectives:** Systemic lupus erythematosus (SLE) is a chronic autoimmune disease with significant morbidity and premature mortality. As patients with SLE often suffer from multiple comorbid conditions, evaluating the overall health burden is critical for improving risk stratification and long-term outcomes. The Charlson Comorbidity Index (CCI) is a widely used tool for quantifying the burden of comorbidity. This systematic review and meta-analysis aimed to assess the prognostic value of the CCI for all-cause mortality in adult patients with SLE. **Methods:** We conducted a systematic review and meta-analysis in accordance with the PRISMA 2020 guidelines. Three databases (PubMed, Embase, and Web of Science) were searched up to May 2025. Three studies (n = 1175 participants) met the inclusion criteria. Eligible studies included adult SLE populations that evaluated the comorbidity burden using the CCI and reported all-cause mortality. Study characteristics and effect sizes were extracted, and a fixed-effects model (after considering both random- and fixed-effects approaches) was applied to calculate pooled odds ratios (ORs). Risk of bias was assessed using the Newcastle–Ottawa Scale. **Results:** Three observational studies (n = 1175 participants) met the inclusion criteria. All demonstrated a significant association between higher CCI scores and increased all-cause mortality. The pooled OR for mortality in patients with a high comorbidity burden was 3.92 (95% CI: 2.74–5.60), with no observed heterogeneity (I^2^ = 0%). The risk of bias was moderate to high across all studies. **Conclusions:** Multimorbidity, as measured by the CCI, is a strong independent predictor of mortality in SLE. Integrating comorbidity assessment into rheumatology care may enhance prognostic evaluation, guide personalized treatment, and support interdisciplinary management strategies for patients with complex disease profiles.

## 1. Introduction

Systemic lupus erythematosus (SLE) is a complex autoimmune disease characterized by multisystem inflammation, a relapsing–remitting clinical course, and significant heterogeneity in its presentation and outcomes [1,2]. Despite advances in immunosuppressive therapies, SLE remains associated with increased morbidity and premature mortality, often driven not only by the disease activity itself but also by coexisting chronic conditions such as cardiovascular disease, renal impairment, infections, and metabolic disorders [3,4,5].

As the global population with SLE ages and survival improves, multimorbidity—the presence of two or more chronic conditions—has emerged as a critical determinant of prognosis and healthcare burden [6,7]. Multimorbidity complicates treatment-related decision-making, increases healthcare utilization, and is strongly linked to poor outcomes, including mortality. While disease-specific indices such as the Systemic Lupus International Collaborating Clinics Damage Index (SLICC-DI) are commonly used in SLE, these tools primarily capture cumulative disease-related damage rather than comorbid conditions unrelated to SLE pathogenesis or treatment [8].

The Charlson Comorbidity Index (CCI) is a widely used and validated scoring system that quantifies the comorbidity burden and predicts mortality across various medical populations [9]. Although initially designed for hospitalized patients, its simplicity and generalizability have led to its adoption in chronic disease research, including rheumatology [10]. However, the prognostic value of the CCI in SLE remains incompletely defined, with studies yielding inconsistent findings. Some have reported a strong association between higher CCI scores and increased mortality risk [11,12], while others have found weaker or non-significant associations [13]. Moreover, there is limited consensus on how best to operationalize comorbidity measurements in SLE, whether through general tools like the CCI or disease-specific instruments.

In this context, the CCI was selected because of its unique strengths. Unlike the SLICC-DI, which focuses on cumulative lupus-specific damage, or the RDCI, which was designed for rheumatic diseases, the CCI provides a standardized, generalizable measure of multimorbidity that allows comparisons across different chronic conditions [13]. Its widespread use, ease of calculation, and incorporation into electronic health record systems make it particularly useful for pragmatic risk stratification in routine clinical practice [11,12]. At the same time, by evaluating the CCI in SLE, this review contributes to the ongoing debate about whether general or disease-specific comorbidity indices best capture prognosis in autoimmune diseases.

Given the rising prevalence of multimorbidity and its potential impact on clinical outcomes in SLE, a comprehensive synthesis of the available evidence is urgently needed. This systematic review and meta-analysis aimed to evaluate the association between comorbidity burden—measured using the CCI—and all-cause mortality in adult patients with SLE. By consolidating existing evidence, this work seeks to clarify the prognostic role of multimorbidity in SLE, inform integrated care approaches in rheumatology, and support risk stratification strategies that incorporate both disease-specific and general health indicators.

## 2. Materials and Methods

### 2.1. Protocol and Registration

This systematic review and meta-analysis adhered to the Preferred Reporting Items for Systematic Reviews and Meta-Analyses (PRISMA) 2020 guidelines [13]. The study protocol was prospectively registered with the International Prospective Register of Systematic Reviews (PROSPERO; registration ID: CRD420251064330).

### 2.2. Eligibility Criteria

We included original observational studies—either retrospective cohort or cross-sectional—that evaluated adult patients (aged ≥ 18 years) diagnosed with systemic lupus erythematosus (SLE) on the basis of established classification criteria such as the American College of Rheumatology (ACR) or the Systemic Lupus International Collaborating Clinics (SLICC) criteria [14]. For quantitative synthesis, eligible studies were required to assess comorbidity burden using the Charlson Comorbidity Index (CCI) and to report all-cause mortality as an outcome with effect estimates (odds ratios [ORs], hazard ratios [HRs]) or sufficient data to compute them. We excluded studies that did not report mortality outcomes, those using comorbidity instruments other than the CCI (e.g., SLICC-DI, RDCI, or simple condition counts), pediatric cohorts, conference abstracts without extractable data, overlapping cohorts, and non-English publications.

The CCI is a validated tool that assigns weighted scores to 17 chronic conditions, including myocardial infarction, congestive heart failure, peripheral vascular disease, cerebrovascular disease, dementia, chronic pulmonary disease, connective tissue disease, peptic ulcer disease, mild liver disease, diabetes mellitus (with and without complications), hemiplegia, moderate or severe renal disease, malignancy, leukemia, lymphoma, moderate or severe liver disease, metastatic solid tumor, and AIDS/HIV [9]. In SLE cohorts, renal disease, diabetes mellitus, cardiovascular disease, cerebrovascular disease, and chronic pulmonary disease were the most frequent comorbidities contributing to higher CCI scores. It is important to note that because SLE itself falls under the category of “connective tissue disease”, most investigators emphasize non-SLE comorbidities when interpreting CCI scores in this population.

### 2.3. Information Sources and Search Strategy

A comprehensive literature search was conducted in PubMed, Embase, and Web of Science from the databases’ inception to May 2025. Search terms included combinations of “systemic lupus erythematosus”, “Charlson Comorbidity Index”, “comorbidity”, “multimorbidity”, “mortality”, “prognosis”, and “survival” to ensure broad coverage of both traditional and contemporary terminology. The complete database-specific search strategies are provided in Supplementary Table S1. In addition, the reference lists of all eligible articles and relevant reviews were manually screened to identify any additional studies not captured by the electronic search.

### 2.4. Study Selection and Data Extraction

Two independent reviewers (RO and RY) screened the titles and abstracts according to the inclusion criteria. Full texts of potentially eligible articles were reviewed in detail. Discrepancies were resolved by discussion or consultation with a third reviewer. Data were extracted using a standardized form and included:

- Study design and location,
- Sample size and demographic characteristics,
- Method of comorbidity assessment (CCI or specific comorbidities),
- Follow-up duration,
- Reported effect sizes (ORs or HRs with 95% CIs),
- Variables adjusted for in multivariable models.

### 2.5. Risk of Bias Assessment

The Newcastle–Ottawa Scale (NOS) was used to assess the risk of bias for each included study [15]. This scale evaluates three domains: the selection of study groups, the comparability of groups, and the ascertainment of the outcome. Scores range from 0 to 9, with studies scoring 6 or higher considered to have moderate to high methodological quality. The results of the quality assessment are summarized in Supplementary Table S1. We also applied the Grading of Recommendations Assessment, Development and Evaluation (GRADE) framework to assess the certainty of evidence for the primary outcome (mortality).

### 2.6. Data Synthesis and Statistical Analysis

For the meta-analysis, both fixed- and random-effects models were considered. Given the absence of heterogeneity across studies (I^2^ = 0%), a fixed-effects model was applied to calculate pooled ORs and 95% confidence intervals (CIs) for all-cause mortality associated with a higher comorbidity burden. A random-effects model was initially considered, but the fixed-effects model was statistically appropriate in this context. Specifically, for the Greenstein et al. study [16], which analyzed the Charlson Comorbidity Index as a continuous variable, we extracted the reported OR per one-point increase in CCI. We recalculated it into a binary equivalent (high vs. low comorbidity burden) using the method described by Chinn (2000) for converting continuous to binary log odds estimates, allowing for comparability across studies [17]. Statistical heterogeneity was assessed using the I^2^ statistic, with thresholds of 25%, 50%, and 75% indicating low, moderate, and high heterogeneity, respectively. Between-study variance (Tau^2^) was also calculated. To evaluate publication bias, funnel plots were generated, and Egger’s test was performed. All statistical analyses were conducted using EZR version 1.51 (Saitama Medical Center, Jichi Medical University, Saitama, Japan), a graphical interface for R (The R Foundation for Statistical Computing, Vienna, Austria) [18].

## 3. Results

### 3.1. Study Selection: PRISMA Flow Diagram

**Figure 1:**
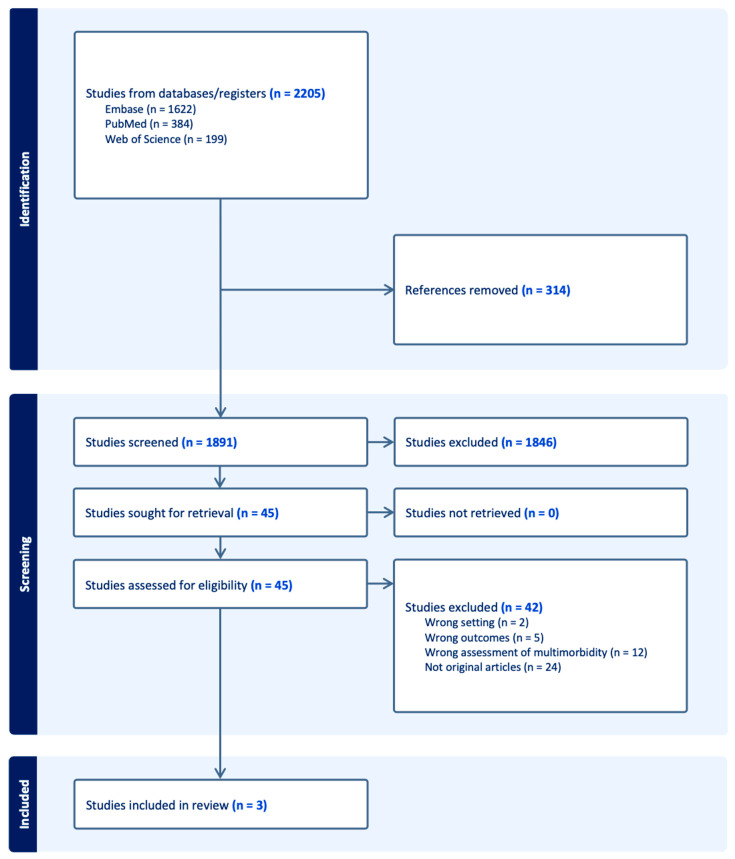
PRISMA Flow Diagram of the Study Selection Process. This flow diagram illustrates the identification, screening, eligibility assessment, and inclusion of studies evaluating the association between the Charlson Comorbidity Index and mortality in patients with systemic lupus erythematosus.

A total of 2205 records were identified through electronic database searches: 1622 from Embase, 384 from PubMed, and 199 from Web of Science. After removing 314 duplicates, 1891 studies were screened by title and abstract. Of these, 45 full-text articles were assessed for eligibility. Thirty-nine were excluded for reasons including an inappropriate setting (n = 1), unrelated outcomes (n = 4), wrong assessment of multimorbidity (n = 12), and non-original articles (n = 24). Ultimately, three studies met the inclusion criteria and were included in the systematic review and meta-analysis. The detailed selection process is illustrated in the PRISMA flow diagram (Figure 1).

### 3.2. Study Characteristics

This meta-analysis included three observational studies published between 2011 and 2019, conducted in Sweden, South Africa, and Singapore. All studies investigated the association between comorbidity burden and all-cause mortality in patients with SLE. Sample sizes ranged from 240 to 673 participants.

The mean age of participants ranged from 32.1 to 47 years, with the proportion of female patients varying from 88% to 92%. All participants were adults diagnosed with SLE on the basis of standardized criteria such as ACR or SLICC classification. Comorbidity burden was consistently evaluated using the CCI. In two studies, the CCI was analyzed categorically with a cutoff of ≥2 to define a high comorbidity burden, while one study (Greenstein et al. [16]) used a continuous CCI score. The CCI accounts for weighted scores assigned to various chronic conditions, including cardiovascular, renal, and metabolic diseases. Across the included studies, the comorbidities most often contributing to elevated CCI scores in SLE patients were renal disease, diabetes mellitus, cardiovascular disease, cerebrovascular disease, and chronic pulmonary disease.

All studies reported all-cause mortality as the primary outcome. Mortality was assessed either in hospital or across defined follow-up periods. Adjustments for potential confounders—such as age, sex, and disease duration—were made using multivariable regression analyses. Table 1 summarizes the study characteristics and patient demographics.

### 3.3. Risk of Bias Within Studies

Risk of bias was assessed using the Newcastle–Ottawa Scale (NOS), applying the appropriate version for retrospective cohort and cross-sectional designs. The three included studies received scores ranging from 6 to 8 out of a maximum of 9 stars, indicating moderate to high methodological quality.

The retrospective cohort studies by Jönsen et al. (2011) [10] and Yang et al. (2014) [19] demonstrated strong performance in the domains of selection and outcome ascertainment; however, both were limited by incomplete adjustment for disease-specific confounders, such as lupus activity indices and immunosuppressive treatments. The nested case–control study by Greenstein et al. (2019) [16] provided sufficient information on sample selection and comorbidity assessment. As a nested design, it allowed for the temporal sequencing of comorbidities and mortality, and offered efficiency in evaluating relatively uncommon outcomes. However, it remains vulnerable to potential selection bias, depending on the control selection, and may be less generalizable than cohort designs.

Common methodological limitations included (1) limited adjustment for clinical factors such as disease activity or immunosuppressive exposure; (2) variability in how the comorbidity burden was operationalized—categorical CCI cutoff vs. continuous CCI score; and (3) differences in follow-up duration, ranging from in-hospital mortality to longitudinal mortality without standardized time frames. Despite these limitations, all three studies met the methodological criteria to be included in the meta-analysis due to their relevance, analytical clarity, and overall quality. A detailed summary of the NOS assessment is provided in Supplementary Table S2.

According to the GRADE assessment, the certainty of the evidence linking higher CCI scores with increased mortality in SLE was rated as low to moderate, primarily due to the observational design, limited number of studies, and risk of residual confounding factors (disease activity, treatment exposure, socioeconomic status).

### 3.4. Results of Individual Studies and Synthesis

All three included studies reported a significant positive association between comorbidity burden, as measured by the CCI, and all-cause mortality in patients with SLE. The CCI was analyzed either categorically (e.g., CCI ≥ 2 vs. <2) or as a continuous score, capturing the impact of comorbid conditions such as cardiovascular disease, diabetes mellitus, and chronic kidney disease on patient outcomes.

The reported effect sizes, expressed as ORs, ranged from 2.1 to 5.3 across individual studies. Despite variation in the operationalization of the CCI and differences in clinical settings (hospitalized vs. mixed populations), all studies consistently demonstrated that a higher comorbidity burden was significantly associated with increased mortality risk. Most studies performed multivariable regression analyses, adjusting for key confounders including age, sex, and disease duration.

**Figure 2:**
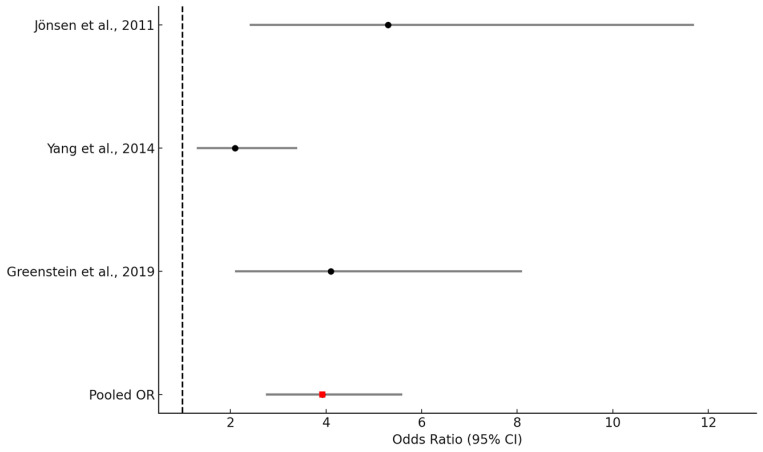
Forest plot of the association between comorbidity burden and all-cause mortality in patients with systemic lupus erythematosus (SLE). Each horizontal line represents the 95% CI of an individual study (Jönsen et al., 2011; Yang et al., 2014; Greenstein et al., 2019) [10,16,19], with the black square indicating the point estimate of the OR. The red diamond represents the pooled OR from the meta-analysis. The dashed vertical line at OR = 1 indicates no association.

**Figure 3:**
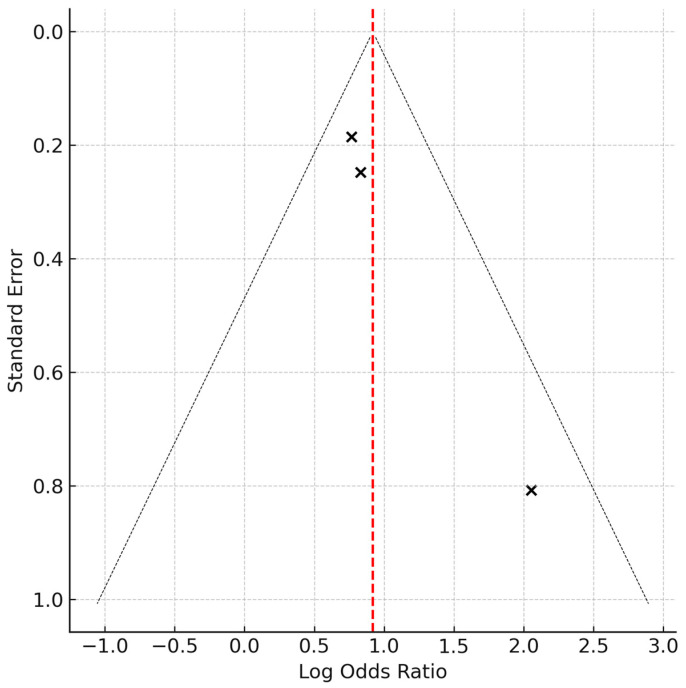
Funnel plot for publication bias assessment.

A fixed-effects meta-analysis yielded a pooled OR of 3.92 (95% CI: 2.74–5.60), indicating a statistically robust and clinically meaningful increase in mortality risk among SLE patients with an elevated comorbidity burden. This summary estimate and the individual study effects are illustrated in Figure 2. Both Egger’s and Begg’s tests were conducted to evaluate publication bias. Egger’s test showed no significant funnel plot asymmetry (*p* = 0.91), and Begg’s test using Kendall’s tau also returned a non-significant result (*p* = 1.00). These findings suggest no evidence of small-study effects or publication bias. This is visually supported by the symmetrical distribution of study points in the funnel plot (Figure 3), reinforcing the robustness of the meta-analytic results.

To test the stability of the pooled estimate, we performed a leave-one-out sensitivity analysis. Sequential exclusion of each study yielded pooled ORs ranging from 3.58 to 4.21, all of which remained statistically significant. These findings suggest that no single study disproportionately influenced the overall association between comorbidity burden and mortality in SLE.

### 3.5. Legend

This forest plot displays the odds ratios (ORs) and 95% confidence intervals (CIs) for the association between comorbidity burden and all-cause mortality in the three included studies. Comorbidity was assessed using the Charlson Comorbidity Index (CCI). A pooled OR was calculated using a fixed-effects model and is represented by a red point. The vertical dashed line at OR = 1 indicates the null hypothesis of no association. As shown in Figure 2, all three included studies demonstrated effect estimates in the same direction with overlapping confidence intervals, supporting the consistency of the association.

This funnel plot illustrates the distribution of individual study effects (log odds ratios) against their standard errors. The vertical red dashed line represents the pooled log odds ratio. The symmetry of data points within the funnel suggests a low risk of publication bias. However, as recommended by PRISMA 2020 and the Cochrane Handbook, a funnel plot’s interpretation is unreliable when fewer than 10 studies are included; therefore, the results should be interpreted with caution.

## 4. Discussion

### 4.1. Summary of Main Findings

This meta-analysis demonstrated a strong and consistent association between multimorbidity and all-cause mortality among patients with systemic lupus erythematosus (SLE). On the basis of three observational studies conducted in geographically and clinically diverse settings, the pooled odds ratio (OR) for mortality among patients with a higher comorbidity burden was 3.92 (95% CI: 2.74–5.60). This indicates that SLE patients with multiple comorbidities have nearly four times greater odds of mortality compared with those with fewer or no comorbid conditions. These findings underscore the importance of extending clinical attention beyond lupus disease activity to include the systematic management of coexisting conditions such as cardiovascular disease, chronic kidney disease, and diabetes mellitus [20,21]. As the SLE population ages, multimorbidity is expected to become more prevalent, highlighting the need for integrated care that bridges rheumatology and general internal medicine [22,23].

### 4.2. Comparison with Previous Literature

While previous studies have acknowledged the prognostic significance of comorbidities in SLE, few have quantitatively synthesized this association [6,24,25,26]. This review integrates data from three countries—Sweden, South Africa, and Singapore—enhancing the generalizability of the findings across various healthcare contexts. Despite differences in how comorbidities were operationalized (e.g., CCI as a binary vs. stratified variable), the effect direction and magnitude remained remarkably consistent, with no observed heterogeneity (I^2^ = 0%).

Compared with SLE-specific instruments like the Systemic Lupus International Collaborating Clinics Damage Index (SLICC-DI) or the Rheumatic Disease Comorbidity Index (RDCI), the CCI offers a broadly applicable, easily implementable method for quantifying overall health burden [27,28,29]. Its widespread use and integration into electronic health records make it a practical tool for risk stratification in both specialized and generalist care settings.

The CCI reflects general multimorbidity, while the SLICC-DI measures cumulative organ damage attributable to lupus and its treatment. Although conceptually related, they are complementary rather than overlapping constructs [27,28,29]. Discordant profiles are clinically standard—for example, patients with a high CCI but a low SLICC-DI, such as older individuals with vascular or metabolic comorbidities, and patients with a low CCI but a high SLICC-DI, such as younger individuals with lupus-related organ damage [28]. Recognizing these distinct patterns may refine risk stratification and guide individualized management in SLE.

### 4.3. Clinical Implications

These findings support the potential utility of structured comorbidity assessment tools, such as the CCI, in the care of SLE patients [30,31]. However, the evidence is based on only three studies of heterogeneous design. Thus, the CCI should be regarded as an associated prognostic marker rather than a definitive independent predictor of mortality. Practical barriers also exist for implementation in routine rheumatology practice, including time constraints, incomplete or inconsistently coded comorbidity data, and limited integration into some electronic health record systems [32,33]. Addressing these barriers will be essential for translating comorbidity assessment into daily clinical workflows.

In addition, publication bias was assessed through both visual (symmetrical funnel plot) and quantitative methods (Egger’s test *p* = 0.91; Begg’s test *p* = 1.00). However, because only three studies were included, these tests lack sufficient statistical power and should be interpreted cautiously, as recommended by PRISMA 2020 and the Cochrane Handbook. All three included studies met a moderate to high standard of methodological quality (Newcastle–Ottawa scale scores of 6–8), affirming the robustness of the pooled findings despite these constraints.

### 4.4. Comparison of Comorbidity Indices in SLE

Several indices exist for assessing comorbidity or multimorbidity in rheumatic diseases. The CCI remains one of the most widely used tools due to its simplicity, validation across diverse populations, and integration into electronic health records [8]. However, the CCI has known limitations that may have influenced our findings. For example, the disproportionately high weight assigned to HIV infection—initially reflecting high mortality in the 1980s—may no longer be appropriate in settings with improved HIV care [29]. In the South African cohort (Greenstein et al., 2019 [16]), where HIV prevalence is relatively high, this weighting could have inflated the CCI scores and potentially overestimated mortality risk [29]. Similarly, because the CCI does not capture lupus-specific factors, such as persistent disease activity, corticosteroid exposure, or cumulative organ damage, it may underestimate risk in patients whose comorbidity burden is primarily driven by lupus [29]. Indices such as the SLICC-DI, which account for disease-specific organ damage, might provide complementary or superior predictive value for long-term outcomes in SLE. Future work directly comparing the CCI with the SLICC-DI across diverse cohorts could clarify the relative strengths and limitations of each tool in prognostic modeling [29]. In contrast, the SLICC-DI is tailored for SLE, capturing cumulative organ damage attributable to both disease activity and treatment [27]. Similarly, the RDCI was explicitly developed for rheumatic diseases, including variables that are more relevant to autoimmune conditions [28].

While SLICC-DI and RDCI may provide greater specificity for lupus-related morbidity, their use is less common in routine care and less standardized across settings [34]. In contrast, the CCI remains a pragmatic tool for general risk stratification, allowing for cross-disease comparisons [16,29]. Therefore, although imperfect, the CCI offers a balance between feasibility and prognostic utility, making it valuable for broad epidemiologic studies and health services research [28]. Moreover, its widespread use enables comparisons between SLE and other chronic diseases, complementing disease-specific tools like the SLICC-DI rather than replacing them. In clinical practice, using the CCI alongside the SLICC-DI may provide a more comprehensive picture of both general multimorbidity and lupus-specific damage, thereby improving holistic risk assessment.

### 4.5. Limitations

This analysis has several important limitations that should be considered when interpreting the findings. First, only three studies met the inclusion criteria, which inevitably limits the statistical power of the meta-analysis and precludes meaningful subgroup analyses (e.g., by age, sex, or geographic region). Consequently, while the pooled estimate is robust across these studies, it may not fully capture heterogeneity in different clinical contexts. Although only three studies were included, the leave-one-out sensitivity analysis demonstrated consistent results (pooled Ors: 3.58–4.21), supporting the robustness of our findings despite the small evidence base.

Second, there was variability in how the comorbidity burden was defined and operationalized. For example, two studies analyzed the CCI using a categorical cutoff (≥2), whereas one used a continuous CCI score. Although we recalculated the continuous effect size for comparability, subtle differences in operationalization may still influence the precision of the pooled estimate. Similarly, the CCI itself has inherent limitations, such as outdated weightings for conditions like HIV and the lack of lupus-specific damage metrics, which may reduce its sensitivity in this population.

Third, the observational nature of all included studies restricts causal inference. While a strong association between a higher comorbidity burden and mortality was consistently observed, residual confounding by unmeasured factors—such as disease activity, medication exposure, and socioeconomic status—cannot be excluded.

Fourth, the included studies primarily relied on cross-sectional or baseline assessments of comorbidity, without capturing longitudinal changes in multimorbidity over time. As a result, the dynamic evolution of comorbidities and their cumulative effects on mortality could not be assessed. In particular, while the Greenstein et al. study [16] benefited from the efficiency and temporal advantages of a nested case–control design, potential selection bias and limited generalizability must be considered when interpreting its results.

Fifth, none of the eligible studies reported both the CCI and SLICC-DI jointly with analyzable statistics. This prevented us from conducting a direct statistical assessment of their correlation or combined predictive performance.

Sixth, only three studies were eligible for inclusion, conducted in Sweden, South Africa, and Singapore. This geographic and ethnic diversity highlights the global relevance of SLE but also restricts generalizability, as significant populations from North America, Latin America, and other parts of Asia were not represented. Therefore, our conclusions should be interpreted with caution until validated in larger, multiethnic cohorts.

Finally, the small number of included studies also limits the reliability of publication bias assessments, as funnel plot asymmetry and statistical tests such as Egger’s or Begg’s have low power when fewer than 10 studies are available. In addition, because our search was restricted to English-language publications, there is a potential for language bias. Relevant studies published in languages other than English may have been overlooked, which could limit the comprehensiveness and global applicability of our findings.

### 4.6. Future Directions

Future research should prioritize longitudinal cohort studies that not only track the evolution of comorbidities but also seek to define a specific CCI threshold that optimizes sensitivity and specificity for predicting mortality in SLE. Establishing such a cutoff could standardize the application of CCI in clinical practice and facilitate its integration into risk stratification algorithms. At the same time, there is a pressing need to develop and validate a novel SLE-specific multimorbidity index that combines the general health burden captured by the CCI with key lupus-related prognostic factors, such as persistent disease activity, treatment-related organ damage, and immunosuppressive exposure. Comparative studies directly evaluating the CCI, SLICC-DI, and any new hybrid indices across diverse, multiethnic cohorts would help clarify their relative and combined value for prognostic modeling. Additionally, implementation science approaches should be used to evaluate the impact of integrating comorbidity assessment into routine clinical workflows on clinical outcomes and healthcare utilization [35,36]. Future prospective cohorts should systematically capture both the CCI and SLICC-DI, which would allow joint analyses and clarify their independent and combined contributions to mortality and other outcomes.

## 5. Conclusions

This meta-analysis suggests that a higher comorbidity burden, as measured by the Charlson Comorbidity Index, is consistently associated with increased all-cause mortality in patients with systemic lupus erythematosus. While causality cannot be inferred and the small number of studies limits generalizability, these findings highlight the potential value of incorporating comorbidity assessment into routine SLE care.

## Figures and Tables

**Table 1:** Summary of study characteristics and patient demographics among included studies evaluating comorbidity and mortality in SLE.

First Author (Year)	Country	Study Design	Sample Size (n)	Mean Age (Years)	Female (%)	Comorbidity Assessment	Follow-Up Duration	Primary Outcome
Jönsen et al. (2011) [10]	Sweden	Retrospective cohort	673	42	90%	CCI (cutoff ≥ 2)	10 years	Mortality
Yang et al. (2014) [19]	Singapore	Retrospective cohort	262	39	88%	CCI (cutoff ≥ 2)	In-hospital	Mortality
Greenstein et al. (2019) [16]	South Africa	Nested case-control	240	32.1	88%	CCI	Median: 5 years	Mortality

Footnote: CCI, Charlson Comorbidity Index. All studies included adult SLE patients diagnosed according to ACR or SLICC classification criteria. A high comorbidity burden was defined as CCI ≥ 2 in Jönsen [10] and Yang [19], and analyzed as a continuous variable in Greenstein.

## Data Availability

No new data were created or analyzed in this study. Data sharing is not applicable to this article, as it is based on previously published studies that are publicly available and properly cited in the reference list. The full search strategies and extracted datasets used in this review are available from the corresponding author upon reasonable request.

## Supplementary Materials

The following supporting information can be downloaded at https://www.mdpi.com/article/10.3390/healthcare13182285/s1. Table S1. Detailed search strategies used in the systematic review (PubMed, Embase, and Web of Science). Table S2. Risk of bias assessment of included studies using the Newcastle–Ottawa scale (NOS).

## Abbreviations

The following abbreviations are used in this manuscript.

SLE	Systemic Lupus Erythematosus
CCI	Charlson Comorbidity Index
OR	Odds Ratio
CI	Confidence Interval
PRISMA	Preferred Reporting Items for Systematic Reviews and Meta-Analyses
PROSPERO	International Prospective Register of Systematic Reviews
ACR	American College of Rheumatology
SLICC	Systemic Lupus International Collaborating Clinics
NOS	Newcastle–Ottawa Scale
HR	Hazard Ratio
SD	Standard Deviation
