# Supplementary material for: Integrating Multimorbidity Assessment into Rheumatology Care: Prognostic Role of the Charlson Comorbidity Index in Systemic Lupus Erythematosus
Source: Healthcare (Basel). 2025 Sep 12;13(18):2285. doi: 10.3390/healthcare13182285 (PMC12470168; doi:10.3390/healthcare13182285)
Supplement: Supplementary file 1 [file healthcare-13-02285-s001.zip › healthcare-3816450-supplementary.pdf]

Table S1. Search Strategies Used in the Systematic Review

Database	Search Strategy	Date of Last Search
PubMed	("systemic lupus erythematosus"[MeSH Terms] OR "systemic lupus erythematosus"[Title/Abstract]) AND ("Charlson Comorbidity Index"[Title/Abstract] OR "comorbidity"[Title/Abstract] OR "multimorbidity"[Title/Abstract]) AND ("mortality"[Title/Abstract] OR "prognosis"[Title/Abstract] OR "survival"[Title/Abstract])	May 15, 2025
Embase	('systemic lupus erythematosus'/exp OR 'systemic lupus erythematosus':ti,ab) AND ('Charlson comorbidity index':ti,ab OR 'comorbidity':ti,ab OR 'multimorbidity':ti,ab) AND ('mortality':ti,ab OR 'prognosis':ti,ab OR 'survival':ti,ab)	May 15, 2025
Web of Science	TS=("systemic lupus erythematosus") AND TS=("Charlson Comorbidity Index" OR "comorbidity" OR "multimorbidity") AND TS=("mortality" OR "prognosis" OR "survival")	May 15, 2025

Table S2. Risk of Bias Assessment of Included Studies Using the Newcastle-Ottawa Scale (NOS)

This table presents the methodological quality evaluation of the six included studies, assessed by the Newcastle-Ottawa Scale. Studies are listed in chronological order from 2011 to 2019.

Study	Study Design	Selection (max 4)	Comparability (max 2)	Outcome/Exposure (max 3)	Total Score (max 9)
Jönsen et al. (2011)	Retrospective cohort	4	1	3	8
Yang et al. (2014)	Retrospective cohort	4	1	2	7
Greenstein et al. (2019)	Cross-sectional	3	1	2	6
